# Modelling and forecasting risk dependence and portfolio VaR for cryptocurrencies

**DOI:** 10.1007/s00181-023-02360-7

**Published:** 2023-01-16

**Authors:** Jie Cheng

**Affiliations:** School of Computing and Mathematics, Keele University, MacKay Building, Keele, ST5 5BG UK

**Keywords:** Cryptocurrencies, Generalized autoregressive score (GAS) model, Multivariate probabilistic forecasts, Portfolio management, G11, G17, C53

## Abstract

In this paper, we investigate the co-dependence and portfolio value-at-risk of cryptocurrencies, with the Bitcoin, Ethereum, Litecoin and Ripple price series from January 2016 to December 2021, covering the crypto crash and pandemic period, using the generalized autoregressive score (GAS) model. We find evidence of strong dependence among the virtual currencies with a dynamic structure. The empirical analysis shows that the GAS model smoothly handles volatility and correlation changes, especially during more volatile periods in the markets. We perform a comprehensive comparison of out-of-sample probabilistic forecasts for a range of financial assets and backtests and the GAS model outperforms the classic DCC (dynamic conditional correlation) GARCH model and provides new insights into multivariate risk measures.

## Introduction

During the last years, cryptocurrencies gain more and more attention not only from ordinary investors but also from regulatory authorities and policy makers. Cryptocurrencies are decentralized currencies that are powered by their users with no central authority and therefore are independent of monetary politics and not controlled by the existing banking system^1^. Bitcoin, the largest cryptocurrencies was created in 2009 and since then numerous other cryptocurrencies have been created. After a stable period of development, most of the cryptocurrencies started to climb and dramatically increased in the period 2016 to 2020 with pricing bubbles in 2018 (Corbet et al. 2018). After that, all major cryptocurrencies’ prices have exhibited tremendous fluctuation with the sharpest drop during March 2020 selloff, as a result of the COVID-19 outbreak.

Existing literature on the cryptocurrencies market includes studies focusing on hedging and safe-haven properties of cryptocurrencies (e.g. Bouri et al. 2017; Conlon and McGee 2020), market efficiency (e.g. Nadarajah and Chu 2017; Tran and Leirvik 2020), volatility patterns and portfolio of cryptocurrency markets (Katsiampa 2017), most of which provide the within-sample fit for univariate cases. On the other hand, to account for the structure linkage and interdependencies among the cryptocurrencies and other financial assets, different multivariate approaches including the GARCH-DCC models (Guesmi et al. 2019; Ghabri et al. 2021), the GARCH-BEKK models (Katsiamp et al. 2019; Stavroyiannis and Babaros 2017) and GARCH-copula models (Bouri et al. 2018; Boako et al. 2019; Syuhada and Hakim 2020) have documented for volatility forecasting and risk management.

While these studies provide useful analyses, they also confirm that both the conditional volatilities and the correlations of the cryptocurrencies change over time, especially during the bubble period in 2018 and the pandemic era in 2020. Therefore, we pay attention to the observation-driven time-varying multivariate generalized autoregressive score (GAS) model to examine the price dependency relationships and portfolio value-at-risk (VaR) of cryptocurrencies; particularly, Bitcoin (BTC), Ethereum (ETH), Litecoin (LTC) and Ripple (XRP) are considered. The generalized autoregressive score-driving model (GAS) is proposed by Creal et al. (2013), and it nests many well-known models, including GARCH (Bollerslev 1986) and ACD (Engle and Russell 1998) models. Tafakori et al. (2018) consider an asymmetric exponential GAS model to predict Australian electricity returns. Chen and Xu (2019) use both univariate and bivariate GAS models to analyse and forecast volatilities and correlations between Brent, WTI and gold prices. To the best of our knowledge, no other study has ever used the multivariate GAS model to forecast the volatility and correlation of cryptocurrencies.

Due to the relatively young literature on cryptocurrency, there are few studies related to out-of-sample forecasting performance for both dependence structure and volatility. Amongst those, Syuhada and Hakim (2020) construct a dependence model through vine copula and provide the value-at-risk (VaR) forecasts. Chi and Hao (2021) show GARCH model’s volatility forecast is better than the option implied volatility using the BTC and ETH prices. In our paper, we conduct out-of-sample forecasting performance for both point forecasts (e.g. VaR) and density forecasts. In order to see how effectively the GAS model treats different dynamic features simultaneously in a unified way, we compare the forecasting results with those of the classic dynamic conditional correlation generalized autoregressive conditional heteroskedasticity (DCC-GARCH) model (Engle 2002).

Our main findings are as follows: First, beside the most applied volatility models, GARCH, asymmetric GARCH specifications including GJR-GARCH and APARCH models are also considered for the univariate ETH, LTC, BTC and XRP return series. Interestingly, the additional parameters in these models, which are supposed to show the asymmetric volatility response to past returns (so-called leverage effect), are not significant for all the cryptocurrencies in this paper. These results are consistent with those found in Chi and Hao (2021) and Syuhada and Hakim (2020). Several studies apply the asymmetric GARCH models to cryptocurrencies’ return series; however, they either use a GARCH-type model with Gaussian innovation (Cheikh et al. 2020) or show rather weak significant additional terms, which are supposed to reflect the asymmetry (Apergis 2021). One possible explanation is that the traders or investors from the cryptocurrency market are different to those from the stock market. Unlike the stock market which is usually dominated by well informed investors, the cryptocurrency market has more uninformed investors, and the volatility asymmetry, which can be traced to trading activity that has been guided by information asymmetry between well informed and uninformed traders in the market (Avramov et al. 2006), is not significant as it did in the stock market.

Second, we find empirical evidence to show that the forecasting ability of the GAS model is better than those of the DCC-GARCH model. More specifically, the GAS model accounts for large price changes in a very natural way when updating the correlations and volatilities over time, especially during extreme events. This is particularly important when we form a portfolio risk and estimate the corresponding VaR forecasts. Through a sequence of statistical tests, our results prefer the GAS model to the DCC-GARCH models in terms of point (volatilities and correlations) forecasts, quantile (value-at-risk) forecasts and density forecasts.

This paper is organized as follows: Section 2 describes the multivariate GAS model and the DCC-GARCH model. Section 3 provides the data source and preliminary analysis. In Sect. 4, we applied the two multivariate models to the daily cryptocurrencies and present the estimation results for the within-sample period. Moreover, we conduct out-of-sample forecasting performance for volatilities, correlations, VaRs and probability distributions for the two models. Section 5 concludes.

## Empirical models

### The multivariate GAS model

Let ${\varvec{r}}_t$ be an *N*-dimensional random vector at time *t* with conditional distribution

1 $$\begin{aligned} {\varvec{r}}_t\vert F_{t-1} \sim p({\varvec{r}}_t,{\varvec{\theta }}_t), \end{aligned}$$

where $F_{t-1}$ contains all the information up to time $t-1$, ${\varvec{\theta }}_t$ is a vector of time-varying parameters depending on $F_{t-1}$ and a set of static parameters ${\varvec{\phi }}$ for all time *t*. The GAS(p,q) model is an observation-driven model, and the time-varying parameters ${\varvec{\theta }}_t$ are governed by the score of the conditional density in (1) and an autoregressive updating equation

2 $$\begin{aligned} {\varvec{\theta }}_{t+1}={\varvec{\kappa }}+\sum _{i=1}^{p}A_i{\varvec{s}}_{t-i+1}+\sum _{j=1}^{q}B_j{\varvec{\theta _{t-j+1}}}, \end{aligned}$$

where ${\varvec{\kappa }}$, *A* and *B* are the coefficient matrices with proper dimensions and ${\varvec{s}}_t$ is the scaled score function

3 $$\begin{aligned} {\varvec{s}}_t={\varvec{S}}_t\nabla _t({\varvec{r}}_t,{\varvec{\theta }}_t), \end{aligned}$$

with

$$\begin{aligned} \nabla _t&=\frac{\partial }{\partial {\varvec{\theta }}_t}p({\varvec{r}}_t,{\varvec{\theta }}_t),\\ {\varvec{S}}_t&=I_t({\varvec{\theta }}_t)^{-\gamma },\\ I_t({\varvec{\theta }}_t)&=E_{t-1}\left[ \nabla _t\nabla _t^{T}\right] =-E_{t-1}\left[ \frac{\partial ^2\log p({\varvec{r}}_t,{\varvec{\theta }}_t)}{\partial {\varvec{\theta }}\partial {\varvec{\theta }}^{T}}\right] , \end{aligned}$$

where the expectation is taken with respect to the conditional distribution in (1). The additional parameter $\gamma$ is fixed. By choosing different values of $\gamma$, the GAS model encompasses some well-known models (e.g. GARCH, ACD and ACM models, see Creal et al. 2013, for a detailed discussion).

In the application, we consider a GAS(1,1) model with $\gamma =0$ and the conditional distribution in (1) follows a multivariate standardized Student-*t* distribution (Ardia et al. 2019). Therefore, the time-varying parameter vector ${\varvec{\theta }}$ (including location $\mu$, scale $\sigma$, correlation $\rho$ and shape $\nu$ parameters) is given by:

$$\begin{aligned} {\varvec{\theta }}_{t+1}={\varvec{\kappa }}+A{\varvec{s}}_t+B{\varvec{\theta }}_t, \end{aligned}$$

and a natural choice for $S_t$ is identity matrix.

### The multivariate DCC-GARCH model

Following Engle (2002), the DCC-GARCH(1,1) model is as follows. Let ${\varvec{r}}_t$ be an N-dimensional random vector at time *t*, we consider

4 $$\begin{aligned} Var({\varvec{r}}_t\vert F_{t-1})=Q_t=D_tR_tD_t, \end{aligned}$$

where $F_{t-1}$ is the information available up to time $t-1$, $D_t$ is a diagonal matrix such that $D_t=\text {diag}(\sqrt{h_{11,t}},\cdots ,\sqrt{h_{nn,t}})$ and $h_{ii,t}$, $i=1,2,\cdots ,N$ is the conditional variance obtained from the univariate model, which is usually GARCH-type model and $R_t$ is the dynamic conditional correlation matrix. More specifically, let

5 $$\begin{aligned} {\varvec{r}}_t&={\varvec{\mu }}_{t-1}+{\varvec{\psi }}_t,\end{aligned}$$

6 $$\begin{aligned} {\varvec{\psi }}_t&=Q_t^{1/2}{\varvec{\varepsilon }}_t, \end{aligned}$$

then the time-varying correlation matrix $Q_t$ can be updated by

$$\begin{aligned} Q_t=(1-a-b)\bar{Q}+a{\varvec{Z}}_{t-1}{\varvec{Z}}_{t-1}^T+bQ_{t-1} \end{aligned}$$

where $\bar{Q}$ is a symmetric time-invariant unconditional covariance matrix and ${\varvec{Z}}_t=D_t^{-1}{\varvec{\varepsilon }}_t$. In our application, we assume ${\varvec{\varepsilon }}_t$ follows a multivariate standardized Student-*t* distribution, as we did in GAS(1,1) model.

## Empirical application

Daily Cryptocurrencies data, Ethereum (ETH), Litecoin (LTC), Bitcoin (BTC) and Ripple (XRP), in US dollars, are obtained from https://www.cryptocompare.com^2^ using a Python script. Our sample period is from 1 January 2016 till 31 December 2021. We split the sample into two parts, a within-sample period from 1 January 2016 to 31 December 2018, which includes a total of 1096 daily prices and out-of-sample period from 1 January 2019 to 31 December 2021. For each of the datasets, the returns $r_t$ of ETH, LTC, BTC and XRP are calculated as

$$ r_t=100\left[ (\log (P_t)-\log (P_{t-1})\right] , $$

where $P_t$ is the daily closing price at time *t*.

**Fig. 1:**
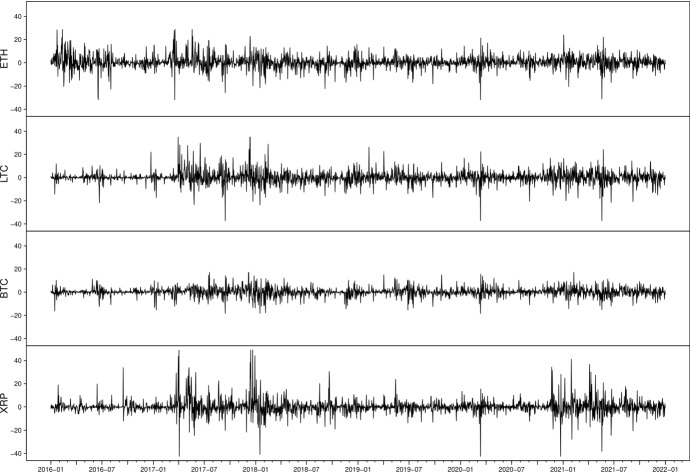
Cryptocurrency return series from January 2016 to December 2021

Cryptocurrency returns are extremely volatile, so we winsorized them at the 0.005% and 99.5% levels. Figure 1 displays the winsorized return series for ETH, LTC, BTC and XRP during the full sample period, i.e. from January 2016 to December 2021. We observe multiple volatile periods for different returns series, but they behave more similarly after 2018. During the March 2020 selloff, all of them experienced the most negative changes. It is worth mentioning that XRP suffered significant price fluctuations during first half of 2021 due to an SEC lawsuit Ripple faced at the end of 2020. Therefore, volatility changes of XRP were mostly caused by updates on the SEC lawsuits after 2021. Table 1 reports the descriptive statistics for the ETH, LTC, BTC and XRP return series. All of them have positive mean returns and leptokurtic empirical distributions for both sample periods. Moreover, the skewness for BTC (XRP) is negative (positive) across the full sample, while ETH and LTC present positive skewness before 2019 and negative one after 2019. For all returns series, the augmented Dickey and Fuller statistics reject the unit root null at 1% significance level, in favour of the stationary time series. The normality is significantly rejected by the enormous Jarque–Bera statistics, indicating the fat-tailed distribution. Engle’s ARCH test (Engle 1982) results reveal the significant ARCH effect, highlighting the application of GARCH-type models.

**Table 1:** Descriptive statistics for ETH, LTC, BTC and XRP return series

Descriptive	In sample				Out-of-sample			
Statistics	ETH	LTC	BTC	XRP	ETH	LTC	BTC	XRP
Mean	0.449	0.181	0.192	0.3255	0.325	0.158	0.255	0.0894
Std.	6.534	5.774	4.016	7.1633	4.843	5.350	3.703	5.9866
Min	$-$31.350	$-$36.358	$-$18.256	$-$42.5689	$-$31.350	$-$36.358	$-$18.256	$-$42.5689
Max	27.802	35.116	17.983	49.0847	23.069	26.873	17.182	41.1113
Skewness	0.228	0.939	$-$0.260	1.7132	$-$0.532	$-$0.531	$-$0.007	0.1702
Kurtosis	6.545	11.362	6.556	16.1768	8.233	9.226	6.256	16.0209
JB stat($\times 10^{3}$)	0.583**	3.354**	0.590**	8.465**	1.302**	1.821**	0.484**	7.748**
ADF(5) stat	$-$14.176**	$-$14.018**	$-$14.305**	$-$11.191**	$-$14.645**	$-$14.923**	$-$14.465**	$-$12.623**
ARCH(5) stat	102.981**	66.626**	92.205**	123.750**	57.904**	41.339**	27.665	72.759**

JB stat, ADF stat and ARCH stat are the statistics testing for normal distribution, stationarity and heteroskedastic effects, respectively. The selected lag number is in parentheses.

*Denote rejections of null hypothesis at 5% significance level.

**Denote rejections of null hypothesis at 1% significance level

**Fig. 2:**
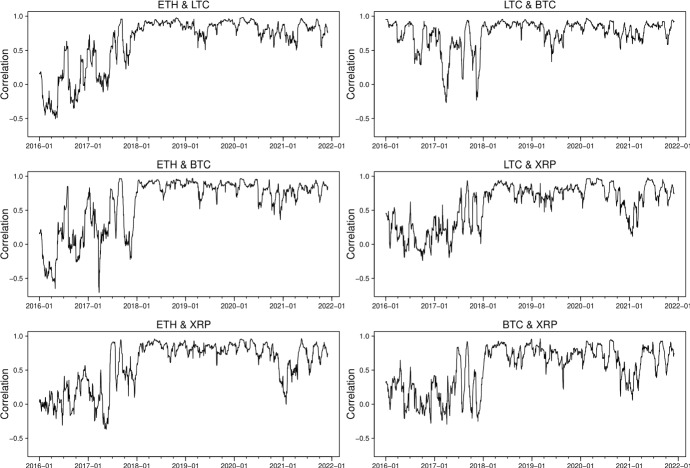
Estimated unconditional correlations (30-day rolling window) from January 2016 to December 2021

Following Tang and Xiong (2012), we first study the full sample rolling unconditional correlations between the ETH, LTC, BTC and XRP return series using a bivariate approach. We rescale the return series by subtracting their means and dividing by their standard deviations and specify the regression of the rescaled return $r_{m,t}^{r}$ on the rescaled return $r_{l,t}^{r}$, with $l,m=1,2,3,4$ and $l\ne m$:

$$ r_{m,t}^{r}=\mu +\tilde{\rho }r_{l,t}^{r}+\eta _t $$

and $\hat{\tilde{\rho }}$ is the estimated unconditional correlation between the two cryptocurrencies returns $r_m$ and $r_l$. The time-varying estimated correlation is obtained by using a rolling window of fix length equal to 30 days. The rolling correlations of full-sample return series are plotted in Fig. 2.

Before 2017, the correlation between BTC and LTC stays high and positive while those between ETH, BTC and XRP are low and negative. This is not surprising as Litecoin was one of the first “altcoins" to draw from Bitcoin’s original open-source code to create a new cryptocurrency, therefore one of the most correlated altcoins with Bitcoin, while Ethereum is launched based on the platform which enables building and deploying smart contracts and decentralized applications, and compete against Bitcoin for market shares; XRP is created as a faster, cheaper, and more energy-efficient digital asset that can process transactions within seconds and consume less energy than some counterpart cryptocurrencies.

From the beginning of 2017 to the middle of 2018, distinct spikes in the correlation can be generally found between the cryptocurrencies. Such spikes may reflect the presence of significant uncertainty during the stage of the development of cryptocurrency market. All the correlations drastically go up at the middle of 2018 and remain positive and strong until the end of the sample. This finding is in line with the current literature (Katsiampa 2019; Katsiampa et al. 2019; Chowdhury et al. 2022; Pace and Rao 2023), and the connectedness between the cryptocurrencies is mainly caused by market uncertainty in response to the 2018 cryptocurrency crash (Aslanidis et al. 2019 and Antonakakis et al. 2019) and the launch on 10 December 2017 of the Bitcoin futures contracts at the Chicago Board Options Exchange (Blau et al. 2020). Moreover, a significant drop in rolling window correlations can be observed at the beginning of 2021 in the cryptocurrency pairs ETH-XRP, LTC-XRP, and BTC-XRP. Again, this is due to the SEC lawsuit Ripple faced. The above bivariate approach considers two return series at a time, as such, cannot exploit the dynamic interdependence simultaneously. To address this issue, we consider the multivariate GAS and DCC models in the next section.

### In-sample results

For notational convenience, let ${\varvec{r}}_t=(r_{1},r_{2},r_{3},r_{4})$ be the returns of the four assets ETH, LTC, BTC and XRP at time *t* and $\rho _{12}$, $\rho _{13}$, $\rho _{14}$, $\rho _{23}$, $\rho _{24}$ and $\rho _{34}$ be the correlation of the return series ETH and LTC, ETH and BTC, ETH and XRP, LTC and BTC, LTC and XRP, and BTC and XRP, respectively. We use the multivariate GAS(1,1) model and the DCC-GARCH(1,1) model (hereafter GAS and DCC) we mentioned in the last section to fit the multivariate return series ${\varvec{r}}_t$, respectively. Based on the fat-tail leptokurtic empirical distributions we obtained in Table 1, the conditional distribution of ${\varvec{r}}_t$ in the GAS model is specified by the multivariate standardized Student-*t* distribution; the univariate and multivariate residuals in the DCC model are also specified by the *t*-distribution.

Asymmetric GARCH specifications including GJR and EGARCH models are also considered for both GAS and DCC models. Interestingly, the additional parameters in these models, which are supposed to show the asymmetric volatility response to past returns (so-called leverage effect), are not significant for all the cryptocurrencies in this paper. These results are consistent with those found in Chi and Hao (2021) and Syuhada and Hakim (2020). Several studies apply the asymmetric GARCH models to cryptocurrencies’ return; however, they either use the GARCH-type model with Gaussian innovation (Cheikh et al. 2020) or show rather weak significant additional terms which are supposed to reflect the asymmetry (Apergis 2021).

**Table 2:** The LR test results for the multivariate GAS model

	Model 1	Model 2	Model 3	Model 4	Model 5
Log LL	$-$12265.63	$-$11682.97	$-$11540.51	$-$11540.80	$-$11533.75
No. of the param	15	17	19	21	23
LR test statistic		1165.32	284.92	0.58	14.10
*p* value		0.000	0.000	0.748	0.001

Model 1 is constant-parameter GAS model, Model 2 indicates time-varying volatility only model, Models 3, 4 and 5 are volatility and correlation time-varying model, volatility, correlation and location time-varying model and all time-varying model. If Model 4 is a constant-location only model, then the LR statistic is 3.62 (*p* value is 0.164)

For the GAS model, the conditional distribution parameters are as follows:

$$ {\varvec{\theta }}=(\mu _1, \mu _2, \mu _3, \mu _4, \sigma _1, \sigma _2, \sigma _3, \sigma _4, \rho _{12}, \rho _{13}, \rho _{14},\rho _{23}, \rho _{24}, \rho _{34}, \nu ) $$

where $(\mu _1, \mu _2, \mu _3, \mu _4)$, $(\sigma _1, \sigma _2, \sigma _3, \sigma _4)$, $(\rho _{12}, \rho _{13}, \rho _{14},\rho _{23}, \rho _{24}, \rho _{34})$, $\nu$ are location, scale/volatility, correlation and shape parameters of the conditional *t*-distribution, respectively. Following (Chen and Xu 2019), we conduct a series of likelihood ratio test (LRT) to see whether these parameters are time varying or not. We are interested in the null hypothesis $H_0: M=M_i$ versus to the alternative hypothesis $H_1: M=M_{i+1}$ for $i=1,2,3,4$, where Model 1 to Models 5 are a series of nested time-varying parameters models, i.e. Model 1 assumes all the parameters are time-invariant, Model 2 is the time-varying volatility-only model and Model 5 is the time-varying volatility, correlation, location and shape model. Clearly, $M_1\subset M_2 \subset M_3 \subset M_4 \subset M_5$, and under the regular conditions, the test statistic LRT shall follow a Chi-square distribution $\chi ^2_k$ with degree of freedom *k* if $H_0$ is true. The LRT test results are listed in Table 2. It is clear that model 5 seems to be a reasonable choice, i.e. the GAS model with time-varying volatility and correlation, location and shape model is used for the return series ${\varvec{r}}_t$ during 2016 to 2019.

**Table 3:** Parameters estimation of the GAS model

Parameters	Estimate	*p* value	Parameters	Unconditional
$\kappa _{\mu _1}$	0.001	0.008	$\mu _1$	0.068
$\kappa _{\mu _2}$	-0.001	0.000	$\mu _2$	-0.075
$\kappa _{\mu _3}$	0.004	0.000	$\mu _3$	0.234
$\kappa _{\mu _4}$	-0.004	0.000	$\mu _4$	-0.243
$\kappa _{\sigma _1}$	0.027	0.000	$\sigma _1$	4.337
$\kappa _{\sigma _2}$	0.014	0.000	$\sigma _2$	2.127
$\kappa _{\sigma _3}$	0.016	0.000	$\sigma _3$	2.374
$\kappa _{\sigma _3}$	0.019	0.000	$\sigma _3$	2.895
$\kappa _{\rho _{12}}$	0.009	0.000	$\rho _{12}$	0.388
$\kappa _{\rho _{13}}$	0.009	0.000	$\rho _{13}$	0.384
$\kappa _{\rho _{14}}$	0.009	0.000	$\rho _{14}$	0.442
$\kappa _{\rho _{23}}$	0.009	0.000	$\rho _{23}$	0.510
$\kappa _{\rho _{24}}$	0.009	0.000	$\rho _{24}$	0.499
$\kappa _{\rho _{34}}$	0.010	0.000	$\rho _{34}$	0.463
$\kappa _{\nu }$	-0.585	0.000	$\nu$	4.000
$a_{\mu }$	0.012	0.000		
$a_{\sigma }$	0.058	0.000		
$a_{\rho }$	0.015	0.000		
$a_{\nu }$	0.414	0.000		
$a_{\mu }$	0.984	0.000		
$a_{\sigma }$	0.981	0.000		
$a_{\rho }$	0.992	0.000		
$a_{\nu }$	0.982	0.000		

This table presents the parameter estimation results for ETH, LTC, BTC and XRP return series during January 2016 to December 2019 (within-sample period). The model assumes time-varying volatility and correlation, location and shape. The unconditional parameters are extracted using identity scaling

The estimation results are presented in Table 3. All the parameters, especially the time-varying parameters of the model (left panel), are significant at the 5% level. We also present the unconditional parameters (right panel) by considering the long-term values of the parameters, i.e. $(I-\hat{B})^{-1}\hat{{\varvec{\kappa }}}$. With regard to the DCC model, similar estimation results are reported in Table 4. The parameters can be divided into two parts, the results of the GARCH model for each individual return series (upper panel) and the dynamic correlation using multivariate *t* distribution (lower panel).

**Fig. 3:**
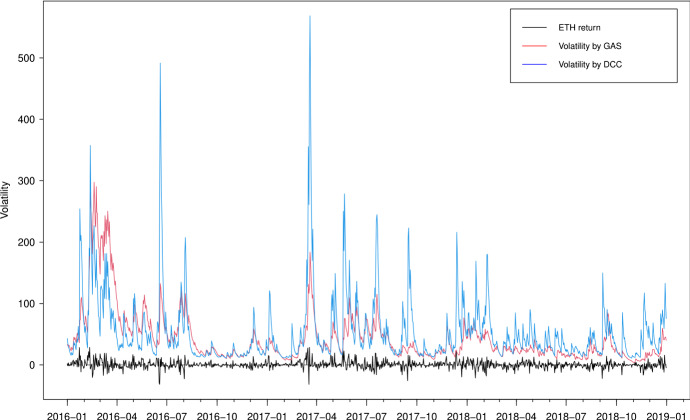
Estimated volatilities of the ETH return using GAS and DCC models

**Fig. 4:**
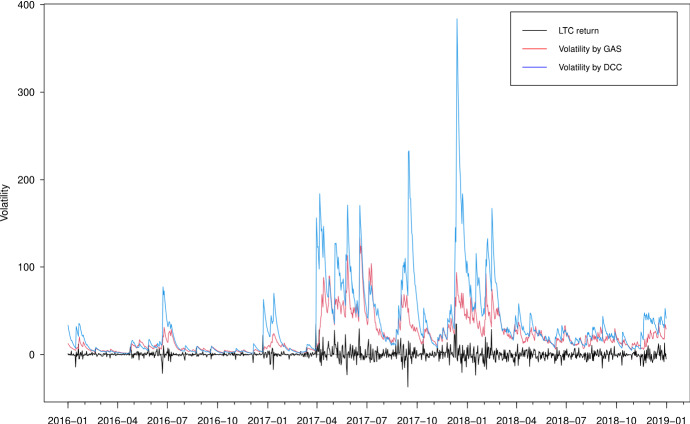
Estimated volatilities of the LTC return using GAS and DCC models

**Fig. 5:**
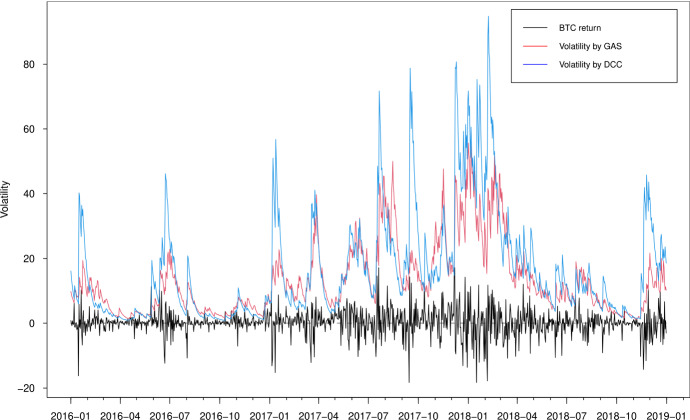
Estimated volatilities of the BTC return using GAS and DCC models

**Fig. 6:**
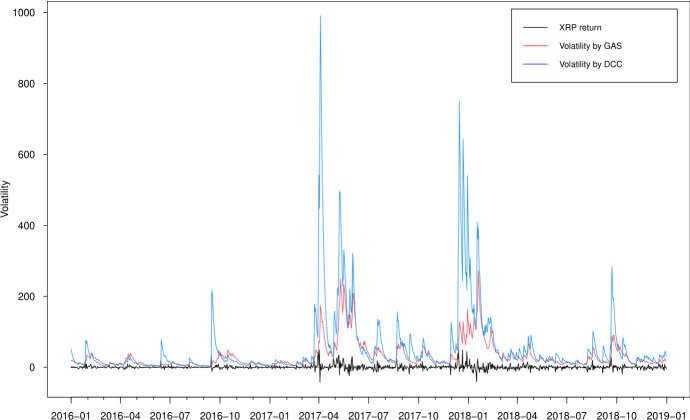
Estimated volatilities of the XRP return using GAS and DCC models

In Figs. 3, 4, 5 and 6, we plot the estimated volatilities for ETH, LTC, BTC and XRP using both GAS and DCC models during the in-sample period, respectively. For all four return series, the DCC model seems to provide more fluctuant volatilities than the GAS model, especially during the 2018 crash period. Clearly, the extreme returns appear to have a strong effect on estimated volatilities for the GARCH models, whereas those for the GAS model appear to be robust.

**Fig. 7:**
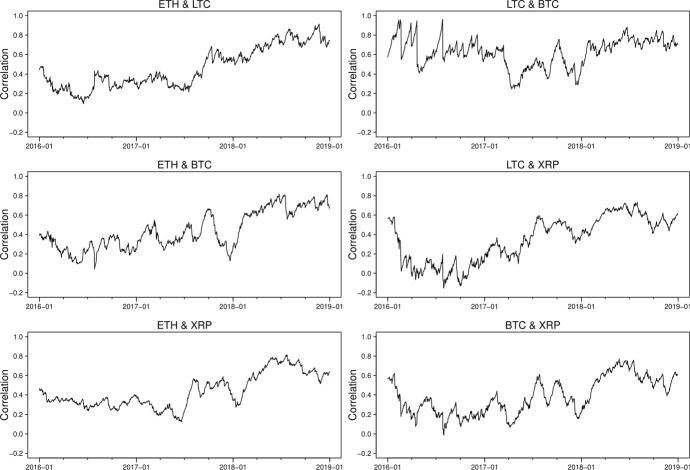
Estimated correlation using GAS models

**Fig. 8:**
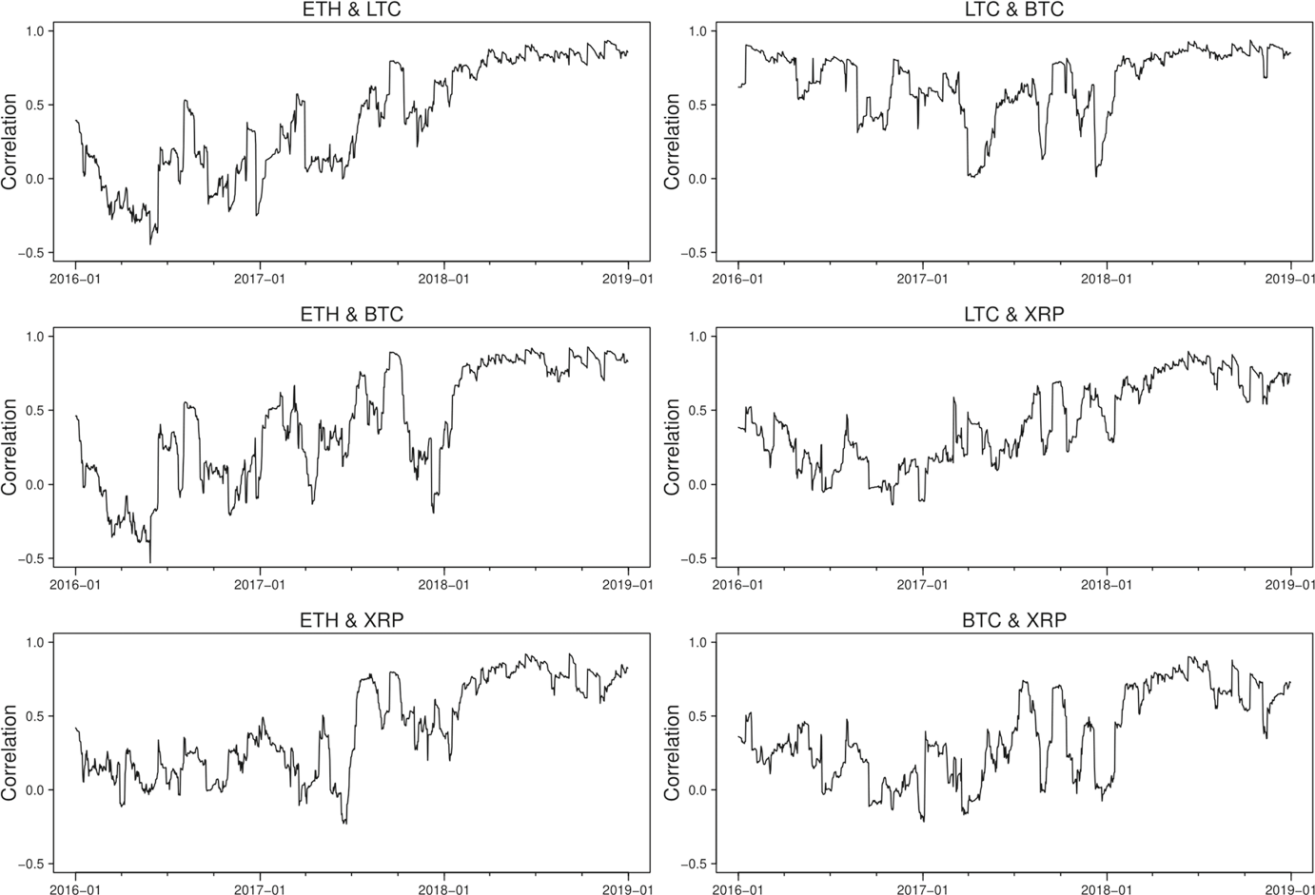
Estimated correlation using DCC models

The correlation estimates from the two models, which are presented in Figs. 7 and 8, show a substantial difference though both models identify a significant persistence of correlations in high positive values between the cryptocurrencies since 2018. The GAS model suggests, in general, positive correlations, varying from -0.15 to 1 between three series, while the DCC model gives correlations fluctuating substantially over time, falling to extreme values around -0.6 during June 2016, which is mainly caused by the instability of the Ethereum prices due to the DAO hack. It is worth noting that the dynamic correlations we derive from DCC multivariate modelling approach appear to be similar to the rolling correlations we estimate in the previously described bivariate setting while those by GAS approach seem to produce more smoothed correlation estimates due to its desirable robust future.

### Out-of-sample results

We now turn to the out-of-sample (OOS) forecast performance of the two models. We compare the one-step-ahead forecasting performance of the GAS model and DCC model using a rolling window scheme. The length of the rolling estimation window is set to be 1096 observations, such that 1096 observations (from January 1 2019, until December 31 2021) are left for out-of-sample forecast evaluation.

**Table 4:** Parameters estimation of the DCC model

Parameters	ETH	*p* value	LTC	*p* value	BTC	*p* value	XRP	*p* value
$\mu$	0.016	0.885	-0.029	0.595	0.200	0.000	-0.274	0.410
$\omega$	2.880	0.055	0.116	0.252	0.077	0.250	0.579	0.012
$\alpha$	0.274	0.000	0.124	0.000	0.128	0.000	0.183	0.000
$\beta$	0.725	0.000	0.874	0.000	0.870	0.000	0.816	0.000
$\nu$	3.301	0.000	3.096	0.000	3.435	0.000	3.000	0.000

Joint parameters		*p* value						
*a*	0.059	0.000						
*b*	0.934	0.000						
$\nu$	4.000	0.000						

This table presents the parameter estimation results for ETH, LTC, BTC and XRP return series during January 2016 to December 2019 (within-sample period). For each individual return series, GARCH model (with *t* innovation) is used and the dynamic correlation is captured using multivariate *t* distribution

#### Volatility and correlation forecast evaluation

To evaluate the forecasting performance of the two models, we construct two measures of realized volatility and correlation using intraday data. The realized volatility is computed as the sum of intraday returns (see, e.g. Andersen et al. (2001)),

7 $$\begin{aligned} RV_t=\sum ^{N_t}_{i=1}r^2_{t,i} \end{aligned}$$

where $r_{t,i}$ is the intraday return on day *t* for intraday period *i* ($i=1,2,\cdots ,N_t$). We use transaction prices of ETH, LTC, BTC and XRP from January 2019 to December 2021, sampled in calendar time and tick-time with 5-minute sampling frequency^3^. The intraday return data are obtained from Bitfinex exchange^4^, using a Python code. The realized correlation^5^ is calculated as:

$$\begin{aligned} RC_{xy,t}=\frac{\sum \nolimits ^{N_t}_{i=1}r_{x,t,i}r_{y,t,i}}{\sqrt{RV_{x,t}}\sqrt{RV_{y,t}}} \end{aligned}$$

where $r_{x,t,i}$ and $r_{y,t,i}$ are the intraday return series for cryptocurrencies *X* and *Y* on day *t* for intraday period *i* ($i=1,2,\cdots ,N_t$) and $RV_{x,t}$ and $RV_{x,t}$ are the realized volatility for *X* and *Y* on day *t*.

**Table 5:** Results of out-of-sample forecasting accuracy

	ETH volatility		LTC volatility		BTC volatility	
	MSE	QLIKE	MSE	QLIKE	MSE	QLIKE
GAS	3960.097	3.624	6888.701	4.121	968.498	4.340
DCC	4135.622	3.679	7034.982	4.145	971.500	4.396
DM	$-$2.292*	$-$0.443	$-$1.686*	$-$0.961	$-$2.817*	$-$0.840

	XRP volatility					
	MSE			QLIKE		
GAS	18960.519			4.272		
DCC	19618.484			4.456		
DM	$-$2.471*			$-$1.892*		

	ETH LTC correlation		ETH BTC correlation		ETH XRP correlation	
	MSE	QLIKE	MSE	QLIKE	MSE	QLIKE
GAS	0.296	0.242	0.229	0.366	0.176	0.372
DCC	0.238	0.261	0.356	0.397	0.292	0.376
DM	$-$1.849*	$-$5.499*	$-$3.252*	$-$3.076*	$-$4.722*	$-$4.731*

	LTC BTC correlation		LTC XRP correlation		BTC XRP correlation	
	MSE	QLIKE	MSE	QLIKE	MSE	QLIKE
GAS	0.188	0.072	0.189	0.116	0.223	0.133
DCC	0.319	0.124	0.244	0.157	0.224	0.147
DM	$-$4.194*	$-$3.007*	$-$2.264*	$-$4.778*	$-$0.010	$-$2.149*

This table presents the t-statistics from Diebold–Mariano (DM) tests of equal predictive accuracy for the rolling window out-of-sample forecasts with different models using corresponding loss functions. A t-statistic greater than 1.96 in absolute value indicates a rejection of the null of equal predictive accuracy at the 0.05 level. These statistics are marked with an asterisk. The sign of the t-statistics indicates which forecast performed better for each loss function: a negative t-statistic indicates that the GAS forecast produced smaller average loss than the DCC forecast, while a positive sign indicates the opposite

Following (Patton 2011), we use two popular and robust loss functions, mean square error (MSE) and Gaussian quasi-likelihood (QLIKE) to compare the forecast accuracy of the GAS and DCC models on the out-of-sample data. These two loss functions are given by,

8 $$\begin{aligned} \text {MSE}_{\sigma ^2}=\frac{1}{N}\sum _{i=1}^{N}\Big (\sigma _i^2-\hat{\sigma }_i^2\Big )^2,\;\;\;\; \text {MSE}_{\rho }=\frac{1}{N}\sum _{i=1}^{N}\Big (\rho -\hat{\rho }_i\Big )^2\;\;\;\; \end{aligned}$$

and

9 $$\begin{aligned} \text {QLIKE}_{\sigma ^2}=\frac{1}{N}\sum _{i=1}^{N}\Bigg (\log (\hat{\sigma }_i^2)+\frac{\sigma _i^2}{\hat{\sigma }_i^2}\Bigg ),\;\;\;\; \text {QLIKE}_{\rho }=\frac{1}{N}\sum _{i=1}^{N}\Bigg (\log (\hat{\rho }_i)+\frac{\rho _i}{\hat{\rho }_i}\Bigg ), \end{aligned}$$

where $\hat{\sigma }_i^2$, $\hat{\rho }_i$ are the rolling forecasts on volatility and correlation of day *i* by the two models, $\sigma _i^2$, $\rho _i$ are the realized volatility and correlation at day *i*, respectively. *N* is the total number of volatility/correlation forecasts. We also use the (Diebold and Mariano 1995) method to test for the null hypothesis that the forecasts by the GAS model are less accuracy than or equal to the forecasts by the DCC model.

Table 5 reports the OOS losses for volatility and correlation, using the loss functions in (8) and (9), for the GAS and DCC models. The Diebold–Mariano statistics on the loss differences are also presented to see whether the gains are statistically significant. Overall, the forecasting ability of volatility and correlation in the GAS model is superior to those of the DCC model. Judging by the MSE and QLIKE, it is significant that the GAS model delivers substantially better correlation forecasts than the DCC model though the two models provide similar correlation forecasts between the BTC and XRP return series in terms of MSE.

**Fig. 9:**
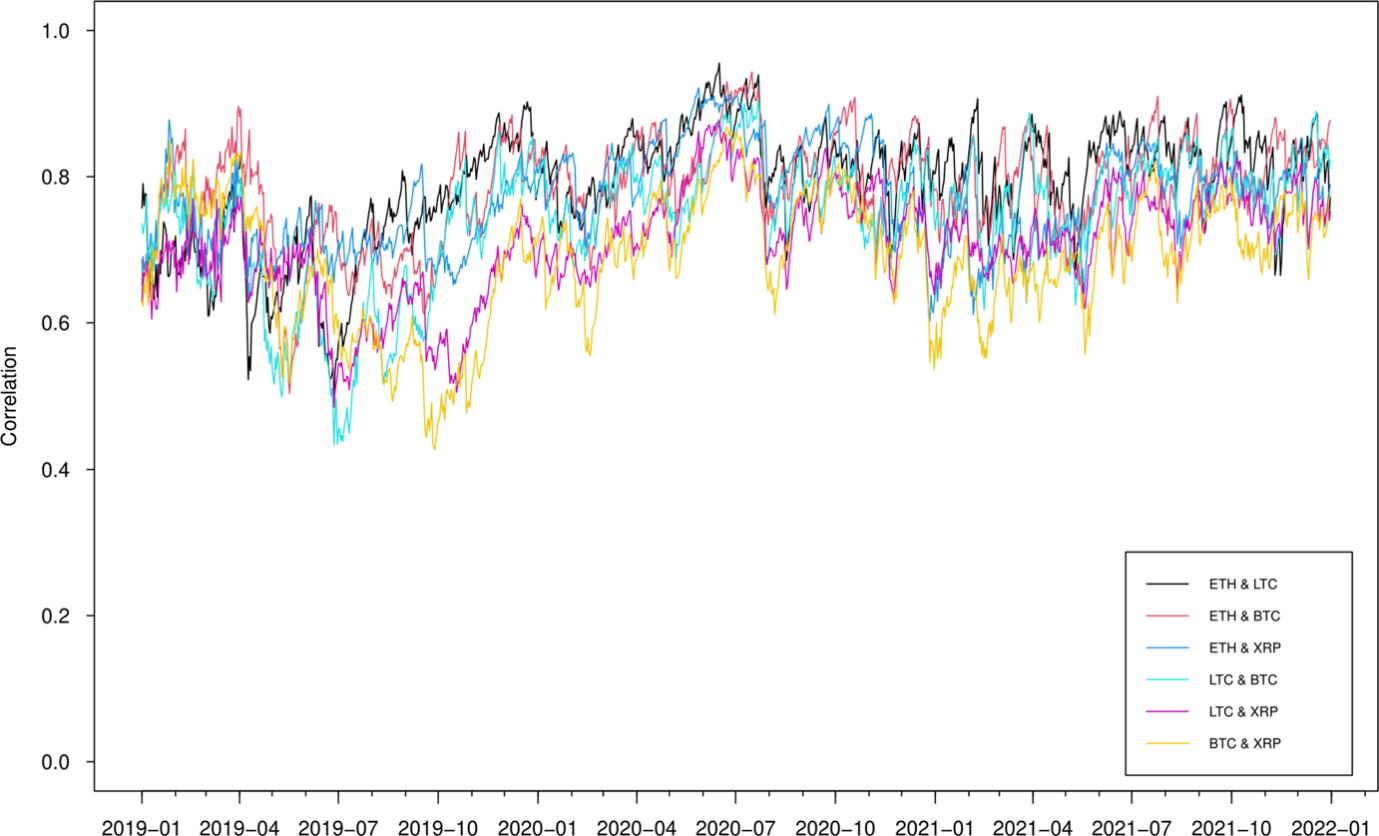
Out-of-sample estimated correlation using GAS models

**Fig. 10:**
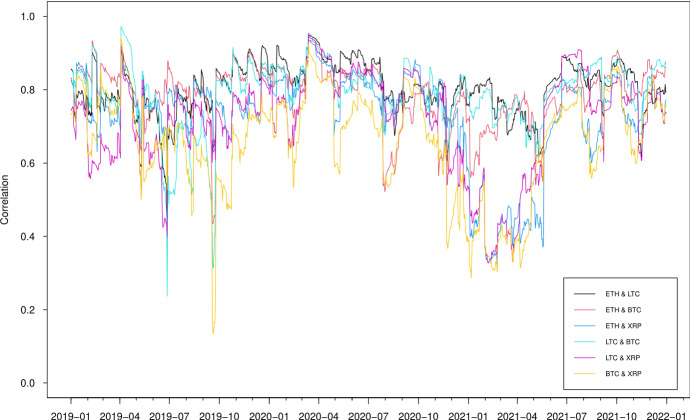
Out-of-sample estimated correlation using DCC models

**Fig. 11:**
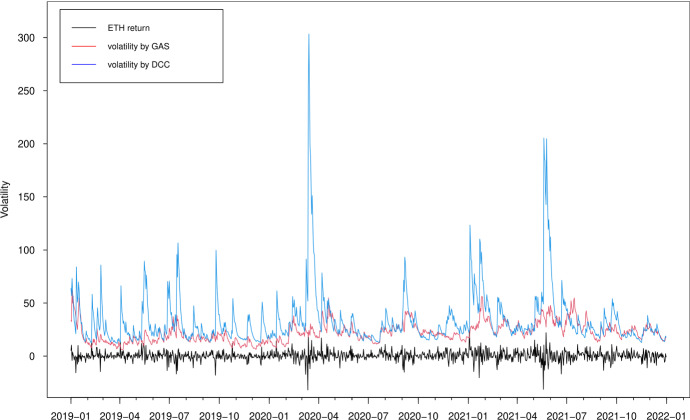
Out-of-sample estimated volatilities of the ETH return using GAS and DCC models

**Fig. 12:**
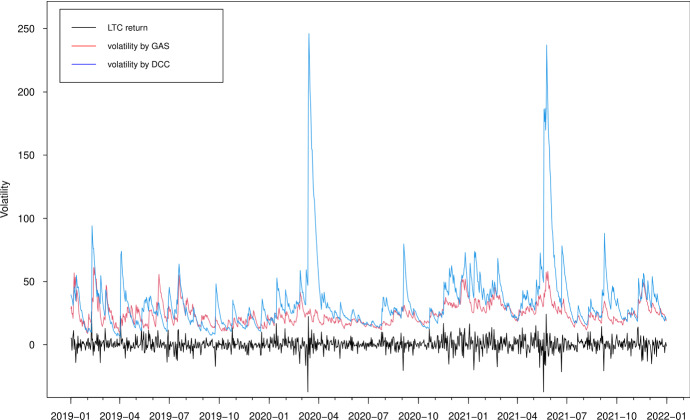
Out-of-sample estimated volatilities of the LTC return using GAS and DCC models

**Fig. 13:**
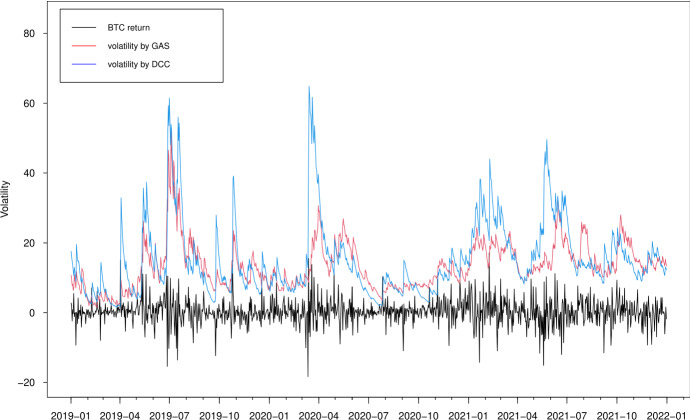
Out-of-sample estimated volatilities of the BTC return using GAS and DCC models

**Fig. 14:**
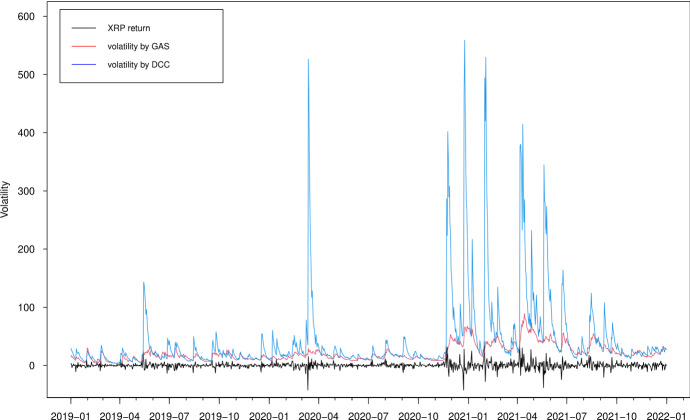
Out-of-sample estimated volatilities of the XRP return using GAS and DCC models

**Fig. 15:**
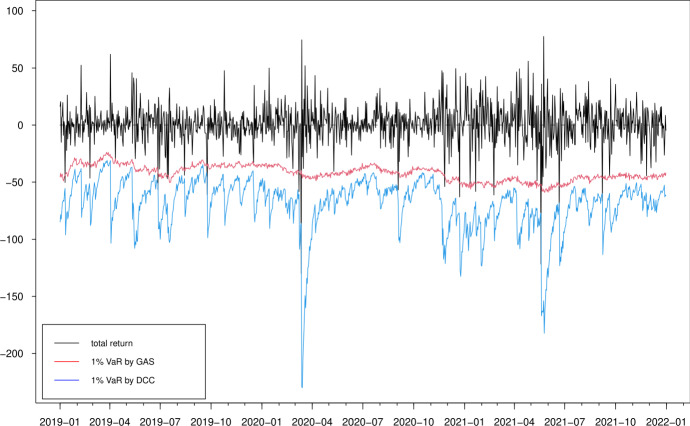
Estimated 1% value-at-risk (VaR) for portfolio 1

**Fig. 16:**
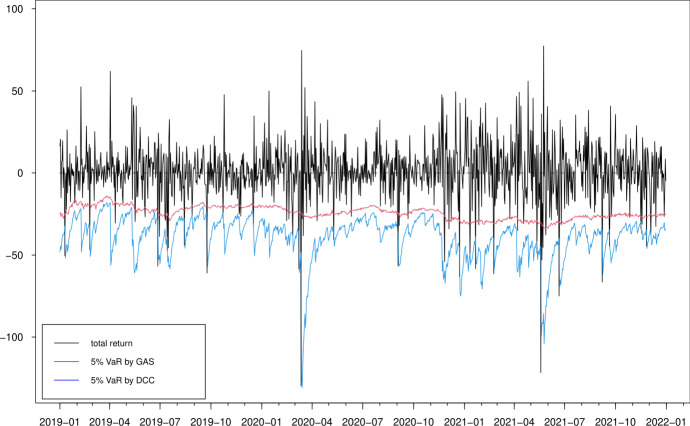
Estimated 5% value-at-risk (VaR) for portfolio 1

The volatility forecasts comparison of MSE and QLIKE between the two models are mixed. The MSE favours the GAS model for all volatilities, while the QLIKE supports the GAS model for XRP volatility only. There is no evidence to show a significant difference of volatility forecasts for ETH, LTC and BTC in terms of QLIKE. These results can be further confirmed in the plots. The difference of correlation forecasts between the two models can be found across the whole OOS period (Figs. 9 and 10), while the volatility forecasts of BTC are similar for both models (Figs. 11, 12, 13 and 14). Noted that the DCC model continuously gives large volatility forecasts for all three return series when there are large changes in the return series.

Interestingly, we find that, on average, for both models, the dynamic correlation forecasts between cryptocurrencies behave similarly in all pairs. The correlations remain positive and at high levels with a few fluctuations across the whole OOS period using GAS model, while those using DCC models gives more sensitive dynamics, especially after January 2020. This could be considered as the consequence of the COVID-19 effect on cryptocurrencies. In particular, during January 2020 to May 2020, weak correlation forecasts can be observed between XRP and other cryptocurrencies using both models, which is, again, due to the SEC lawsuit.

#### Density forecast evaluation

To conduct further the comparison experiment, we use the estimated results for each of the models in the previous section to get one-step-ahead density forecasts and the evaluation is based on scoring rules, which are widely used in weather and climate prediction (Palmer 2012) and financial risk management (Groen et al. 2013). Let ${\textbf {y}}=(y^{(1)},\cdots ,y^{(N)})$ be an observation of the *N*-dimensional random vector, let *f*(.) denote a forecast density of ${\textbf {y}}$, let $\Omega$ denote the set of possible values of ${\textbf {y}}$, and let $\mathcal {F}$ denote a convex class of probability distribution on $\Omega$. A scoring rule is a loss function:

$$\begin{aligned} S(f,y):\mathcal {F}\times \Omega \rightarrow \mathbb {R}\cup \{\infty \} \end{aligned}$$

such that better forecast yields a lower score. A scoring rule *S* is said to be proper if the expected score is optimized, while the true distribution of the observation is issued as a forecast, i.e.

10 $$\begin{aligned} \mathbb {E}_gS(g,\cdot )\le \mathbb {E}_gS(f,\cdot ) \end{aligned}$$

for all $f,g\in \mathcal {F}$. Furthermore, a scoring rule is called strictly proper if equality (10) holds only if $f=g$.

A natural approach is the logarithmic score (Good 1952; Mitchell and Hall 2005; Amisano and Giacomini 2007), which is defined as:

11 $$\begin{aligned} Log S(f,y)=-\log f({\textbf {y}}). \end{aligned}$$

However, the logarithmic score is not sensitive to distance, which means it only rewards the predictive densities for assigning high probabilities to realized values but not the neighbourhood values. To overcome this problem, (Gneiting and Raftery 2007) introduce the energy score which is a generalization of the univariate continuous ranked probability score (CRPS) and allows for a direct comparison of density forecasts. The energy score is defined as:

12 $$\begin{aligned} ES(f,y)=E\left( \Vert Y-{\textbf {y}}\Vert ^{\beta }\right) -\frac{1}{2}E\left( \Vert Y-\tilde{Y}\Vert ^{\beta }\right) \end{aligned}$$

where $\tilde{Y}$ is an independent copy of *Y*, so it is drawn independently from the same distribution *f*(.) as *Y*, $\Vert .\Vert$ is the Euclidean norm. Gneiting and Raftery (2007) show that the energy score is strictly proper with $\beta \in (0,2)$. In application, $\beta =1$ seems to be a standard choice and the score is usually calculated through Monte Carlo methods.

Pinson and Tastu (2013) show that the discrimination ability of energy score may be limited, while the dependence structure of multivariate probabilistic forecasts is misspecified. To overcome this problem, Scheuerer and Hamill (2015) propose the variogram score which is based on pairwise differences:

13 $$\begin{aligned} VS(f,y)=\sum _{i,j=1}^{N}w_{ij}\left( \vert y_i-y_j\vert ^p-E\vert x_i-x_j\vert ^p\right) ^2 \end{aligned}$$

where *N* is the dimension of random vector ${\textbf {y}}$, $x_i$ and $x_j$ are the *i*th and *j*th component of a random vector ${\textbf {x}}$ that is from the distribution *f*, $w_{ij}$ are nonnegative weights that allows one to emphasize pairs of component combinations and standard choice for weights is $w_{ij}=1$. $p>0$ is the order of the variogram score. The variogram score is proper relative to the class of distributions for which the 2*p*-th moments of all elements are finite and it is not strictly proper (Scheuerer and Hamill 2015). In application, the choice of *p* is a trade-off between all relative moments of the pairwise deviation and outliers. Typical choices of *p* include 0.5 and 1.

To test the null hypothesis of equal predictive ability of two competing models based on a given scoring rule, we consider (Diebold and Mariano 1995) type tests using score difference. Given a scoring rule *S*, the score difference is defined as:

$$\begin{aligned} d_{t}=S(\hat{f}_{1},{\textbf {y}}_{t})-S(\hat{f}_{2},{\textbf {y}}_{t}) \end{aligned}$$

where $\hat{f}_{1}$ and $\hat{f}_{2}$ are the density forecasts. The null hypothesis of equal scores is:

$$\begin{aligned} H_0: E(d_{t})=0, \text { for all }t \end{aligned}$$

versus the alternative $H_1: E(d_{t})\ne 0$. It can be shown that, under the null hypothesis, with certain conditions (e.g. see Giacomini and White 2006), the statistic

14 $$\begin{aligned} DM=\frac{\bar{d}}{\sqrt{\hat{\sigma }^2/n}} \rightarrow N(0,1) \end{aligned}$$

where *n* is the forecast sample size, $\bar{d}=\frac{1}{n}\sum _{t=1}^{n}d_t$ and $\hat{\sigma }^2$ is a heteroskedasticity and autocorrelation-consistent variance estimator of $\sigma ^2=var(\sqrt{n}\bar{d})$.

We applied the above three scores to evaluate and compare the density forecasts by GAS and DCC models. For variogram score, we present the results with different *p* values ($p=0.5, 1, 2$) as used in Scheuerer and Hamill (2015)). The overall density forecast can be evaluated using average score $\bar{d}$ during the whole out-of-sample period^6^ and the DM statistics are obtained using the log score in (11), the energy score in (12) and the variogram score in (13). The score difference $d_{t}$ is computed by subtracting the score of the DCC model density forecast from the score of the GAS density forecast, such that negative values of $d_{t}$ indicate the better predictive ability of the forecast method based on the GAS model. Table 6 shows the average score differences $\bar{d}_{n}$ with the accompanying tests of equal predictive accuracy as in (14). These results clearly demonstrate that both energy and variogram scoring rules suggest superior density predictive ability of the GAS model. The large values of average variogram score difference with $p=2$ are caused by the nature of quadratic form, and the results are in accord with the simulation studies by Scheuerer and Hamill (2015).

From the risk management point of view, it is also important to focus on the performance of density forecasts in the region of interest. Therefore, we compare the models in terms of correctly forecasting the 1% and 5% value-at-risk (VaR) at 1-day horizons for both individual cryptocurrencies and different portfolios that can be constructed from the three cryptocurrencies. We define five different arbitrary portfolios, $p_{jt}=g_jr_t$ for given $4\times 1$ weight vectors $g_j$ and for $j=1,2,3,4,5$. By ordering the cryptocurrencies as ETH, LTC, BTC and XRP, we construct the following long-only and long-short portfolios: $g_1=(1/4,1/4,1/4,1/4)$, $g_2=(1/4,1/4,1/4,-1/4)$, $g_3=(1/4,1/4,-1/4,1/4)$, $g_4=(1/4,-1/4,1/4,1/4)$ and $g_5=(-1/4,1/4,1/4,1/4)$. The long-short positions reflect the relative value bets among these cryptocurrencies.

We simulate 10000 sample paths for ${\textbf {r}}_{t+1}=(r_1,r_2,r_3,r_4)'$, denoted by ${\textbf {r}}_{t+1}^s$ for $s=1,2,\cdots ,10000$ using the multivariate *t* distribution by the GAS and DCC models. We then construct the simulated individual returns $r^s_{i,t+1}$ for $i=1,2,3,4$ and portfolio returns $p^s_{j,t+1}=g_j'{} {\textbf {r}}_{t+1}^s$ for $j=1,2,3,4,5$. We use the sample of 10000 simulated paths to estimate the quantiles of the forecasting distribution at the 1-day horizon. The out-of-sample VaR accuracy is assessed through the unconditional coverage (UC) test (Kupiec 1995) and the conditional coverage (CC) test (Christoffersen 1998).

**Table 6:** Average score differences and tests of equal predictive accuracy

Scoring rule		multicolumn1l$\bar{d}$	multicolumn1lDM test stat.	*p* value
Log score		0.004	0.114	0.454
Energy score		$-$ 0.198	$-$ 6.506	0.000
Variogram score	$p=0.5$	$-$ 1.742	$-$ 6.380	0.000
Variogram score	$p=1$	$-$ 31.980	$-$ 3.882	0.000
Variogram score	$p=2$	$-$ 38247.031	$-$ 4.355	0.000

The table presents the average score difference $\bar{d}^*$ and the corresponding test statistics (with *p* values in the parentheses) for the log score in (11), the energy score in (12) and the variogram score in (13). The variogram scores are presented with $p=0.5, 1$ and 2. The score difference $d_{t}$ is computed for density forecasts obtained from a GAS model with multivariate *t* innovations relative to the DCC model with same innovations, for daily ETH, LTC, BTC and XRP returns over the evaluation period 1 January 2019–31 December 2021

**Table 7:** Results of out-of-sample VaR forecasting performance

		5% VaR		1% VaR	
		UC test	CC test	UC test	CC test
ETH	GAS	9.192 (0.003)	10.310 (0.006)	0.362 (0.547)	2.522 (0.283)
	DCC	44.968 (0.000)	45.331 (0.000)	15.223 (0.000)	15.224 (0.000)
LTC	GAS	2.268 (0.132)	3.293 (0.193)	6.055 (0.014)	6.841 (0.033)
	DCC	24.624 (0.000)	25.070 (0.000)	8.204 (0.004)	8.221 (0.016)
BTC	GAS	16.057 (0.000)	16.068 (0.000)	3.826 (0.051)	4.428 (0.109)
	DCC	14.036 (0.000)	14.075 (0.000)	0.891 (0.345)	1.009 (0.604)
XRP	GAS	2.678 (0.102)	9.890 (0.007)	2.713 (0.099)	2.779 (0.249)
	DCC	24.624 (0.000)	25.611 (0.000)	15.012 (0.000)	17.141 (0.000)
Portfolio 1	GAS	2.268 (0.132)	2.563 (0.278)	1.349 (0.245)	1.766 (0.414)
	DCC	42.233 (0.000)	42.649 (0.000)	8.204 (0.004)	8.221 (0.016)
Portfolio 2	GAS	13.312 (0.000)	14.538 (0.000)	3.826 (0.050)	4.911 (0.086)
	DCC	28.401 (0.000)	29.060 (0.000)	5.901 (0.015)	5.930 (0.052)
Portfolio 3	GAS	1.891 (0.169)	2.123 (0.346)	2.878 (0.090)	4.137 (0.126)
	DCC	30.432 (0.000)	31.176 (0.000)	8.204 (0.004)	8.221 (0.016)
Portfolio 4	GAS	3.592 (0.058)	5.183 (0.075)	8.692 (0.003)	9.238 (0.010)
	DCC	28.400 (0.000)	29.060 (0.000)	11.189 (0.001)	11.196 (0.003)
Portfolio 5	GAS	7.745 (0.005)	9.179 (0.010)	1.658 (0.198)	1.748 (0.417)
	DCC	22.871 (0.000)	23.947 (0.000)	8.691 (0.003)	9.594 (0.008)

The one-step-ahead VaR forecasts for both individual ETH, LTC, BTC and XRP returns and arbitrary portfolios are found simultaneously based on simulated innovation. By ordering the cryptocurrencies as ETH, LTC, BTC and XRP, portfolios 1, 2, 3, 4 and 5 are constructed using weight vectors $g_1=(1/4,1/4,1/4,1/4)$, $g_2=(1/4,1/4,1/4,-1/4)$, $g_3=(1/4,1/4,-1/4,1/4)$, $g_4=(1/4,-1/4,1/4,1/4)$ and $g_5=(-1/4,1/4,1/4,1/4)$, respectively. The column labelled UC and CC reports the unconditional coverage test of Kupiec (1995) and the conditional coverage test of Christoffersen (1998) with *p* values in the parentheses, respectively

Table 7 presents the UC and CC test statistics and the corresponding *p* values of the 5% and 1% VaR forecasts for both individual returns (upper panel) and four portfolios (lower panel). For the individual VaR forecasts, all results, except for BTC returns series, suggest that GAS model performs better than DCC model at the 1% and 5% quantile levels. The GAS and DCC models provide same results for the BTC return: the 1% VaRs forecasts perform reasonably well, but the 5% VaR forecasts are rejected for both tests. Meanwhile, the GAS model outperforms the DCC model in general for all portfolios in the forecasting experiment. The only exception is the portfolio with weights $g_2=(1/4,1/4,1/4,-1/4)$ and $g_5=(-1/4,1/4,1/4,1/4)$ at the 5% significant level and the portfolio with weight $g_4=(1/4,-1/4,1/4,1/4)$ at the 1% significant level, for which both model perform poorly.

In Figs. 15 and 16, we show the 1% and 5% VaR estimates against the realized returns for portfolio 1, i.e. the long-only portfolio with equal weights for the four cryptocurrencies. We observe that typically the VaR estimates based on the DCC models are more extreme, confirming that the DCC model significantly overestimates the risk at both 5% and 1% quantile levels, especially when the return changes are large (e.g. April 2020 and May 2021). These results are in accordance with previous findings (Creal et al. 2011). The estimates of the DCC model are based on lagged squared returns and the forecasts thus move stochastically every day. However, the updating equation in the GAS model with the Student-*t* density provides a more moderate increase in the variance/correlation for a large absolute realization of return. The forecasts using the GAS model naturally inherit the return information. Overall, we conclude that the GAS model has better out-of-sample forecasting behavior.

## Conclusion

We have investigated the co-dependence and portfolio VaR of cryptocurrencies using four popular virtual currencies (Bitcoin, Ethereum, Litecoin and Ripple). The results of the multivariate GAS model show strong dynamic interdependence among the cryptocurrencies throughout the sample period. Our out-of-sample forecasting period notably included the COVID-19 outbreak period, which lasted from early 2020 to the end of 2021. Thus, it sheds new light on the multivariate risk measures of cryptocurrencies for global investors.

We examine the out-of-sample predictive performance of the multivariate GAS model for a range of financial assets at various quantile levels. Using a battery of scoring rules and backtesting procedures, our results show that the GAS model significantly outperforms the traditional DCC-GARCH model. These results still hold if different cryptocurrencies are considered. There is plenty of room for future research on the analysis of cryptocurrencies, especially during financial turmoil. We can extend the existing scoring rules (especially in multivariate cases) to a more flexible form to cover a particular region of the density. An alternative extension could explore the safe-haven properties of cryptocurrencies, stablecoins and traditional assets. Under this framework, the dynamic correlations and the portfolio diversification can be studied systematically.

## Footnotes

1 Since 2019, China’s central bank has announced that all transactions of cryptocurrencies are illegal, effectively banning digital tokens such as Bitcoin. As one of the consequences, the price of major cryptocurrencies has dropped sharply.

2 CryptoCompare’s real-time aggregate index methodology (CCCAGG) calculates the market price of cryptocurrency pairs traded across exchanges. Aggregating transaction data from more than 250 exchanges, CryptoCompare uses a 24-hour volume-weighted average for every currency pair.

3 Liu et al. (2015) finds that it is difficult to significantly beat the realized volatility (RV) using 5-minute intervals.

4 we downloaded the intraday data from different exchanges (e.g. Bitstamp, Coinbase) and the results using these data are the same.

5 Andersen et al. (2001) introduced the realized covariance and the realized correlation comes from the realized covariance divided by the square roots of the realized volatilities in (7).

6 Noted that all the scores we discussed above are proper (Gneiting and Raftery 2007), which means that any incorrect density forecasts $\hat{f}_t$ do not receive a lower average score (negatively oriented score) than the true density.
